# Molecular mechanisms of extrachromosomal circular DNA formation

**DOI:** 10.1093/nar/gkaf122

**Published:** 2025-03-04

**Authors:** Rasmus A B Eugen-Olsen, Judith M Hariprakash, Vibe H Oestergaard, Birgitte Regenberg

**Affiliations:** Department of Biology, University of Copenhagen, Copenhagen, DK-2200 Copenhagen N, Denmark; Department of Biology, University of Copenhagen, Copenhagen, DK-2200 Copenhagen N, Denmark; Department of Biology, University of Copenhagen, Copenhagen, DK-2200 Copenhagen N, Denmark; Department of Biology, University of Copenhagen, Copenhagen, DK-2200 Copenhagen N, Denmark

## Abstract

Recent research reveals that eukaryotic genomes form circular DNA from all parts of their genome, some large enough to carry whole genes. In organisms like yeast and in human cancers, it is often observed that extrachromosomal circular DNA (eccDNA) benefits the individual cell by providing resources for rapid cellular growth. However, our comprehension of eccDNA remains incomplete, primarily due to their transient nature. Early studies suggest they arise when DNA breaks and is subsequently repaired incorrectly. In this review, we provide an overview of the evidence for molecular mechanisms that lead to eccDNA formation in human cancers and yeast, focusing on nonhomologous end joining, alternative end joining, and homologous recombination repair pathways. Furthermore, we present hypotheses in the form of molecular eccDNA formation models and consider cellular conditions which may affect eccDNA generation. Finally, we discuss the framework for future experimental evidence.

## Graphical Abstract

**Graphical Abstract:**
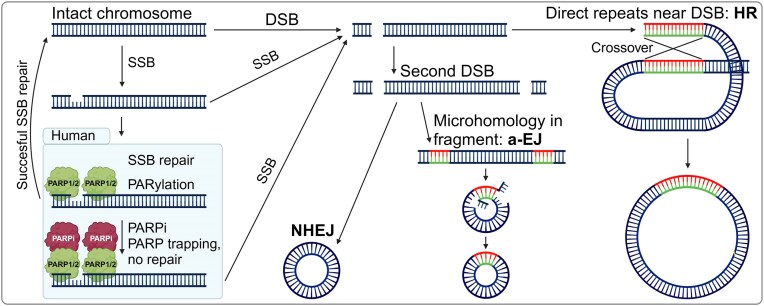

## Introduction

### Extrachromosomal circular DNA and DNA damage

Until recently, it was commonly assumed that a chromosomal deletion would prompt degradation of the deleted fragment, and the potential for the excised DNA to be maintained by the cell was therefore not considered [1]. However, it has since become clear that circularization of a deleted sequence can prevent its loss from the host cell. Genome wide screening for circular DNA revealed that circular DNA was common, and parallel studies in yeast and human showed that deletion can indeed be followed by formation of corresponding circular DNA [2–4]. Circular DNA of chromosomal origin is now known to be found in all studied eukaryotic organisms, and global screens have uncovered that circular DNA can arise from all parts of the tested genomes [5–8]. It is commonly known as extrachromosomal circular DNA (eccDNA) (see Glossary) but can also be referred to as ecDNA, double minutes, and microDNA, among other functional names; for a discussion of nomenclature, see [9, 10]. Found in animals, fungi, and plants in both somatic and germline tissues, eccDNA ranges in size from a hundred base pairs (bp) to a few megabases and can thereby carry whole genes [11–14].

Multiple factors are thought to influence cellular eccDNA copy number and loads, including formation, replication, segregation, elimination, and selection [15, 16]. Once a circle has formed, its potential to be maintained is increased if it is able to replicate. The ability for large circles to replicate is hypothesized to rely on the presence of a replication sequence in the form of a replication origin in humans or an autonomous replication sequence [17, 18] in yeast.

The lack of centromeres on eccDNA leads to uneven segregation [19]. This results in heterogeneous eccDNA copy numbers in daughter cells, and allows genes to amplify to multiple copies per cell within a few cell divisions [14]. The increased copy number enables eccDNA to be transcribed at higher levels than chromosomal DNA, an effect which is further amplified by its accessible chromatin structure and lack of higher-order compaction [20]. These characteristics of eccDNA have significant evolutionary implications [17].

In unicellular organisms, the ability for eccDNA to rapidly modify gene copy numbers and thereby provide accelerated adaptation can provide increased fitness for the host and population. Research on *Saccharomyces cerevisiae* indicates that nutrient scarcity often favours cells with transporter genes on eccDNA [2, 18]. This provides evidence that eccDNA which confers advantageous phenotypes can be selected for and increase in copy number, thereby demonstrating selection as another key phenomenon influencing cellular eccDNA loads.

However, in multicellular organisms, the potential for eccDNA to improve fitness for one cell often comes at a cost for the host organism as a whole. In humans, eccDNA is primarily known from its association with cancer [15], where it mediates the amplification of oncogenes in tumors [19], accelerating tumor evolution [14]. Furthermore, the uneven segregation of eccDNA increases the heterogeneity of tumor cell phenotypes [21], complicating treatment regimens. Lowering eccDNA loads therefore holds clinical potential for cancer treatment. eccDNA formation is hypothesized to be one of the main determinants of cellular eccDNA levels [17].

Therefore, understanding the formation of eccDNA is crucial to unravelling its functionality and potential implications. Though the mechanistic models behind its formation are preliminary, eccDNA is proposed to arise through DNA repairs following DNA damage from replication errors, translocation bridge amplification, and genome-shattering events (chromothripsis), to name a few [15].

DNA damage can be caused by exposure to exogenous mutagenic agents or through endogenous cellular processes. Multiple types of DNA damage can arise, ranging in scope from single-base mutations to severe events like the shattering of entire chromosomes (chromothripsis). Correspondingly, a variety of DNA repair pathways have evolved to handle the different degrees of damage. Besides their canonical role in maintaining genome integrity, DNA repair pathways are hypothesized to occasionally result in eccDNA formation. Mismatch repair (MMR), which is responsible for correcting single nucleotide mutations and small insertions and deletions (indels), is suggested to form eccDNAs with sizes typically <1000 bp [22]. However, most models which are generalizable to the formation of all sizes of eccDNA rely on DNA double-strand break (DSB) repair pathways. While DNA DSBs are serious and relatively infrequent forms of DNA damage, they nonetheless occur regularly, with an estimated 50 DSBs per cell cycle in an average human cell [23]. While eccDNA formation is often overlooked during discussion of DNA misrepair products, links between DSBs and circle formation have been demonstrated in *S. cerevisiae* [5] and human kidney cell lines [24]. Additionally, it has been demonstrated that chromosomal sequences can circularize through CRISPR-Cas9-induced DSBs [3]. However, the extent and manner in which each DSB repair (DSBR) mechanism contributes to eccDNA formation is not well understood.

This review focuses on the link between DSBR and eccDNA formation. We propose models for how DSBR could generate eccDNA, review the evidence for each model, and suggest experimental testing approaches. Our focus spans two organisms: *Saccharomyces cerevisiae* (from here on ‘yeast’) and *Homo sapiens* (from here on ‘human’). Yeast is widely used in biotechnology and is a standard model organism for studying biology, including eccDNA, while humans exhibit eccDNA with clinical significance, particularly in cancer [15]. Although other mechanisms beyond DNA repair may potentially also contribute to eccDNA formation [25], these are beyond this review’s scope and will only be briefly discussed. Furthermore, our main focus is on somatic tissue, although we briefly discuss the formation of eccDNA during meiosis.

### DSBR mechanisms and proposed eccDNA formation models

**Figure 1.**
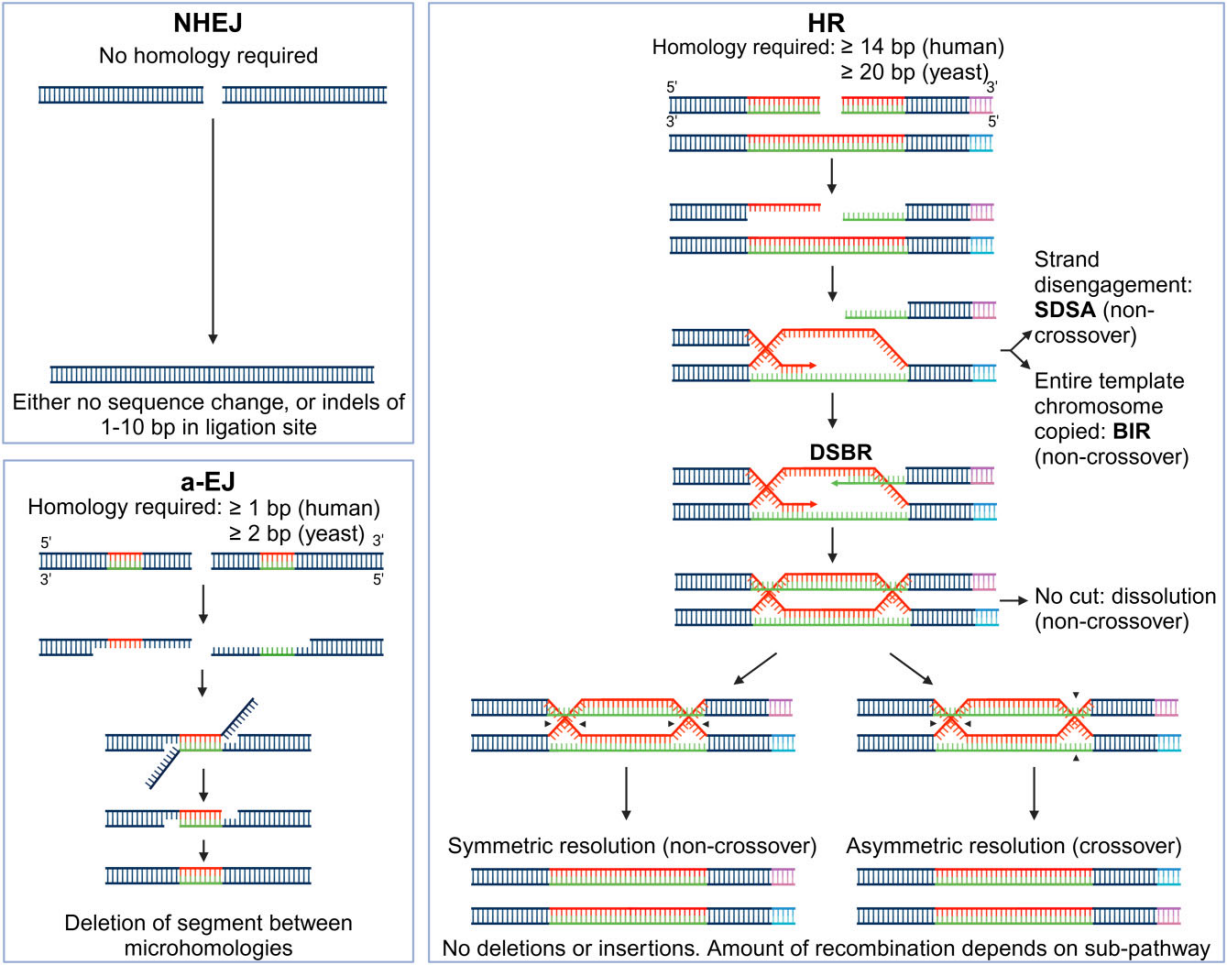
Overview of the three main eukaryotic DSBR mechanisms. NHEJ requires no homology and ligates two pieces of DNA, either with no sequence change or small indels. a-EJ requires short stretches of homology revealed by either short or long-range resection. Microhomologies then anneal, and intermittent sequences protrude as flaps, which are enzymatically removed, after which gap synthesis and ligation complete the repair process. The final product always contains a deletion corresponding to the sequence between the microhomologies. In human a-EJ, insertions may also be present. HR starts with a long-range resection. Revealed single-stranded DNA (ssDNA) can undergo homology search, either in a sister chromatid, a homologous chromosome, or on the same chromosome. Following the annealing of homologies, DNA is synthesized using the invaded sequence as a template. Disengagement of the invading strand leads to repair through synthesis-dependent strand annealing (SDSA), while continued synthesis without second-end capture leads to a copying of the entire chromosome and loss of heterozygosity through the break-induced repair (BIR) pathway. If synthesis is followed by second-end capture, a double Holliday junction (dHJ) is formed. If junctions are dissolved or enzymatically resolved in a symmetric orientation, repair products will be non-crossovers. If dHJ are resolved asymmetrically, products will be crossovers. Figure inspired by [33]. The fourth main DSBR mechanism, single-strand annealing (SSA), is not depicted, as it is not included in our models (see the ‘Model limitations and other sources of eccDNA’ section). Created in BioRender. Eugen-Olsen, R. (2025) https://BioRender.com/i33k341.

To maintain chromosome integrity, organisms have developed multiple DSBR mechanisms, which differ in requirements, accuracy, and activity across cell cycle stages [26, 27]. The primary eukaryotic DSBR pathways include nonhomologous end joining (NHEJ) [28], homologous recombination (HR) [29], and alternative end joining (a-EJ) [30] (Fig. 1). This section will outline how NHEJ, HR, and a-EJ function, and how each pathway can impact eccDNA formation. We have included broad mechanistic descriptions of each pathway to provide an overview for the reader, but note that only selected key steps and proteins are included in our descriptions and models, and outstanding and detailed reviews have been published on each pathway. For deeper insights into each repair mechanism, we recommend the following reviews for yeast NHEJ [31], human NHEJ [28, 32], HR [29, 33], end resection [34], yeast a-EJ, also known as microhomology-mediated end joining (MMEJ) [30, 35], and human a-EJ, also known as polymerase theta-mediated end joining/Polθ-mediated end joining (TMEJ) [36]. We then integrate the molecular interactions of each DSBR pathway with predominant eccDNA formation models from the literature, thus providing a framework for developing hypotheses that can be used to experimentally test each model. Where protein names differ between organisms, yeast proteins are mentioned first with a ^y^ superscript and human proteins with an ^h^ superscript.

### Nonhomologous end joining

NHEJ is a versatile DSBR pathway, as it indiscriminately ligates two ends of double-stranded DNA (dsDNA) without homology requirements. In rare events where DSB ends contain no overhangs, blunt-end ligation occurs with no sequence changes; otherwise, end-processing is required, during which indels of around 1–10 bp may be introduced before sequence ligation [3, 37]. Due to this and the risk of chromosomal rearrangement caused by joining random DNA segments, NHEJ is generally considered error-prone [27, 32]. While NHEJ in yeast and humans share similarities, the frequency of NHEJ usage and some of the proteins involved differ. In yeast, NHEJ is suppressed in the presence of a homologous chromosome or sister chromatid; consequently, in diploids (most frequent natural ploidy) [38], NHEJ is constitutively suppressed, while in haploids, NHEJ is active during the G1 phase [31, 39]. Conversely, NHEJ serves as the primary DSBR mechanism in mammals, predominant and functional across all of interphase [28, 32] but suppressed during M phase [40].

### NHEJ mechanism and proteins involved

NHEJ-mediated DSBR begins with the rapid binding of the heterodimer Ku (Yku70^y^-Yku80^y^ [41]; Ku70^h^-Ku80^h^) and the MRX^y^ (MRN^h^) Complex [42] to the DSB ends, preventing extensive resection [43]. Following this, NHEJ factors (Dnl4p-Lif1p^y^ and Nej1p^y^; XRCC4^h^, XLF^h^, and DNA-PKcs^h^) [44–48] are recruited to the site, forming the core complex. DNA ends may be clean and directly suitable for blunt-end ligation (Fig. 1), but usually contain complex lesions such as overhangs or adducts requiring end processing before ligation. End processing proceeds iteratively [28], depending upon the damage sustained, with the core complex being flexible to accommodate the process at varying positions. Limited resection and synthesis during end processing can result in small indels in the repaired junction, usually in the range of ≤10 bp [49]. Notably, yeast is more efficient at annealing complementary ssDNA overhangs than blunt end-ligation [50]. Once DNA ends have been gathered and potential end processing has occurred, ends are ligated by Dnl4p^y^ [51] (Lig4^h^), completing repair.

### Model for eccDNA formation by NHEJ

A DSB splits a chromosome into two parts, providing a potential substrate for NHEJ. As telomeres inhibit self-ligation [52], NHEJ can only ligate the two free ends created by the DSB, restoring the chromosome.

However, if two intrachromosomal DSBs co-occur, a free fragment is created between the breaks. This presents three options for NHEJ [3]:

- Each free fragment end can be ligated back to its original chromosomal neighbor, restoring the chromosome with its original synteny.
- The free fragment can be flipped before it is ligated to the chromosome, restoring the chromosome with an inversion.
- The ends of the free fragment can self-ligate, and the two chromosomal fragments can be repaired, producing an eccDNA and a chromosome with a corresponding deletion.

**Figure 2.**
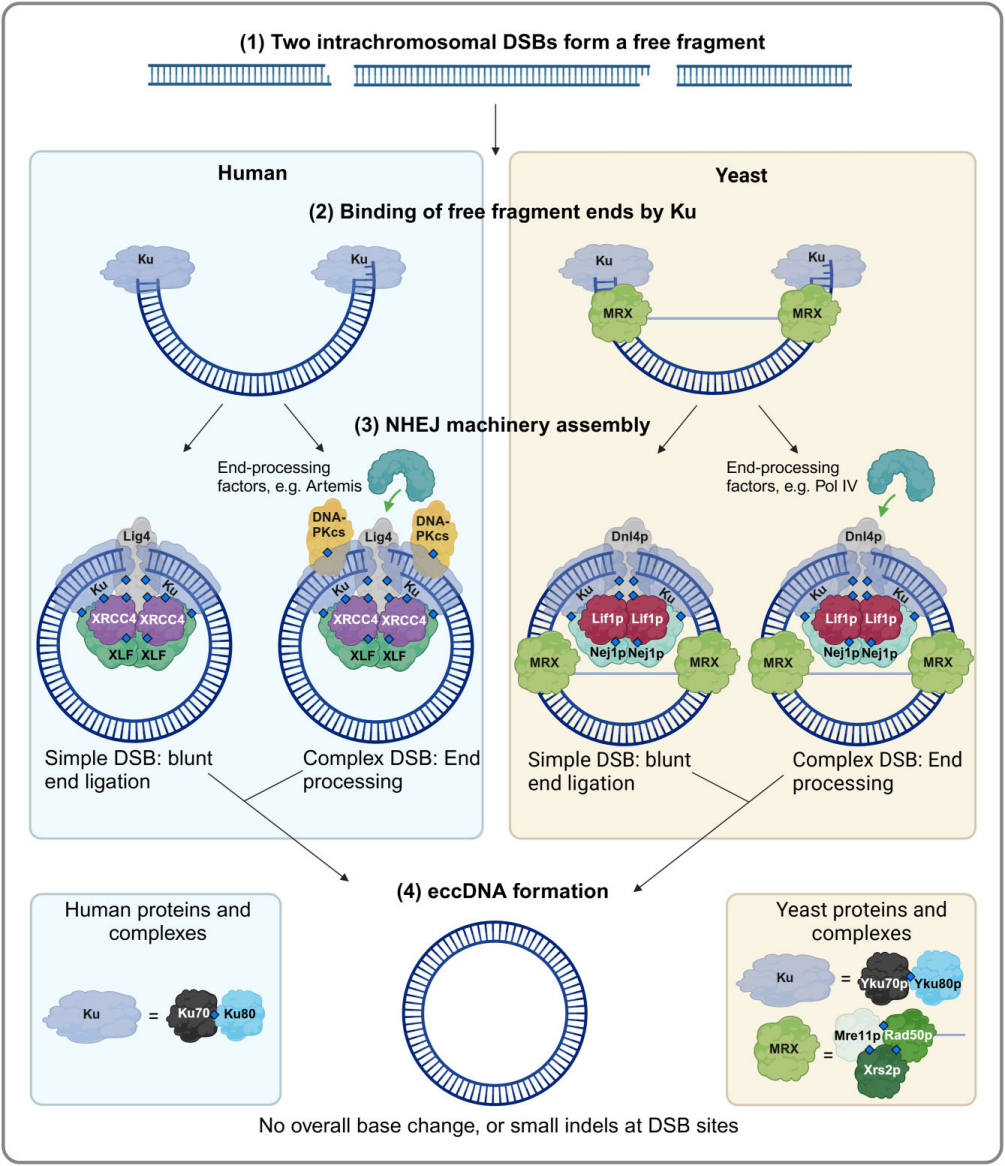
Proposed model for eccDNA generation through NHEJ, based on a model from literature [3] and the protein interactions occurring during regular NHEJ repair of DSBs. The positioning of the proteins is meant to illustrate where they might be located. Diamonds indicate experimentally verified protein bindings. Straight arrows indicate steps in the model, and curved arrows indicate protein involvement. Created in BioRender. Eugen-Olsen, R. (2025) https://BioRender.com/r87d788.

We propose that NHEJ can generate eccDNA from any loci, as it is unrestricted by homology requirements. However, the model necessitates two simultaneous DSBs on the same chromosome, which could be a rare scenario unless the two breaks are mechanistically linked. Class switch recombination in B cells works in exactly this way, and is perhaps the best characterized mechanism leading to production of an eccDNA, which is known as an excision circle [53]. NHEJ is a fast repair mechanism, normally completing the repair of a DSB within 30 minutes in human cells [54]. eccDNA formation thus requires the two DSBs to occur within this timeframe. While the repair of multiple co-occurring DSBs can also lead to translocations through the NHEJ pathway, the 3D arrangement of chromosomes into separated domains [55] could present an intuitive framework for the favourability of eccDNA formation over translocations, where NHEJ has to act across domain barriers. Fig. 2 illustrates the proposed model for NHEJ-mediated eccDNA generation.

### Evidence for NHEJ in eccDNA generation

Indirect evidence of eccDNA formation through NHEJ can be observed when chromosomal fragments form eccDNA without discernible homology [56, 57]. A general screening of yeast eccDNA using the standardized Circle-Seq protocol found that 81% of circles contained breakpoints formed from chromosomal donor sites carrying <7 bp homology [5].

A study of human colon cancer cell lines found increased NHEJ protein expression in cells containing large, gene-carrying eccDNAs. Furthermore, both inhibition and deletion of DNA-PKcs was found to decrease cellular load of these eccDNAs, as well as *DHFR* amplification [58]. In a mouse study [12], the knockdown of key NHEJ factor DNL4 reduced the potential for major satellite DNA to generate eccDNAs, thus underscoring the importance of NHEJ in facilitating the generation of this type of eccDNA. In a study investigating circularization of chromosomal fragments created by two surrounding CRISPR-Cas9 induced DSBs (CRISPR-C), it was found that breakpoints of the generated eccDNAs contained either no or small indels, suggesting formation through NHEJ [3].

NHEJ has also been suggested as an inhibitor of eccDNA formation [24], as Paulsen *et al.* observed that deletion of key NHEJ factors in human and chicken cell lines lead to an increase in small eccDNA load. However, an alternative explanation of this observation could be that knocking out NHEJ proteins downstream of Ku prolongs DSB duration, causing genotoxic stress. When NHEJ is inhibited, the free fragment created by two intrachromosomal DSBs could drift away from the chromosome, preventing its ligation to its original chromosomal position. If Ku disengages from the fragment after it has been spatially separated from the chromosome, another repair pathway, such as a-EJ, could potentially ligate the fragment into an eccDNA. Indeed, a paper studying the effect of NHEJ inhibition on the repair of DSBs generated by CRISPR-C suggests that delaying NHEJ repair could increase the diffusion of the fragment away from the break and favour self-ligation and eccDNA formation [59].

As previously mentioned, NHEJ is active in yeast only in haploid cells during the G1 phase, unlike in humans, where it is active throughout interphase. Therefore, we expect that eccDNA formed by yeast NHEJ will be created during G1, while it may be the default generation mechanism during interphase and in postmitotic tissue in humans.

### Homologous recombination

HR repairs DSBs by synthesizing new DNA using a homologous template (Fig. 1). HR covers multiple sub-pathways, including BIR, SDSA, and DSBR. They all commence with dsDNA resection at the DSB, revealing ssDNA, which is then used to locate and invade a homologous template for annealing followed by DNA synthesis. Sub-pathways mechanisms diverge at this point [29, 33]. In DSBR, synthesis and second-end capture forms a dHJ, which can be resolved to yield crossover or noncrossover between the invading and the template strand [33]. This review will focus on HR in the form of DSBR with crossovers, as we regard this sub-pathway to have the highest potential for eccDNA generation.

HR requires the longest homology and involves the most extensive 5′ resection of the three repair mechanisms discussed here. HR is considered high fidelity and allows error-free DNA repair without indels [33]. Its efficiency depends on homology length and search distance [60].

The frequency of HR usage differs between yeast and humans; cell cycle and ploidy regulate HR in yeast. When a sister chromatid or homologous chromosome is available, yeast predominantly uses HR for DSBR. When neither is present, such as in haploids during G1, NHEJ is preferred to HR [26, 29]. In humans, HR mainly operates during meiosis and the S and G2 phases of mitosis, where a sister chromatid serves as a template. Consequently, HR is less frequently utilized in humans and occurs primarily in mitotically active cells [61]. A lowered level of mammalian HR between homologous chromosomes may persist outside of S and G2, as observed in murine embryonic stem cells [62] and human cancer cells mutated in HR regulators [63].

### HR mechanism and proteins involved

To repair a DSB via HR, the sequence adjacent to the DSB must find a homologous region to invade and use as a repair template (Fig. 1). This requires exonuclease-mediated resection of the DSB dsDNA, resulting in the exposure of ssDNA.

The MRX^y^ (MRN^h^ recruited by ATM^h^ in humans) complex, along with Sae2p^y^ (CtIP^h^), initiates HR by forming a nick near the DSB site, followed by 3′–5′ short-range resection to create ssDNA [64–66]. The exposed ssDNA then acts as an entry point for long-range 5′–3′ resection facilitated by Exo1p^y^ (EXO1^h^) and/or the Dna2p-Sgs1p^y^ (DNA2-BLM^h^/WRN^h^) complex [34].

Rad51p^y^/RAD51^h^ proteins are loaded onto the ssDNA by mediator proteins such as Rad52p^y^ (BRCA2^h^) [67–69]. This forms a nucleoprotein filament that facilitates the search for homologous sequences. Estimates of the minimum homology length required for HR are 20 bp in yeast [70] and 14 bp in humans [71]. Efficiency drastically increases with longer sequence homology in both species [70, 71]. Once homology is found, it forms a D-loop with the invading ssDNA, allowing DNA synthesis and invading strand elongation by polymerase δ (Pol δ) or polymerase ϵ (Pol ϵ) [26, 72]. Following the extension, the repair follows either SDSA, BIR, or DSBR [29, 33]. In SDSA, the invading strand disengages before second-end capture can occur. Though SDSA is favoured in mitotic cells, a lowered level of DSBR persists [73]. In DSBR, the invading strand does not disengage: after synthesis, second-end capture between the second DSB end and the D-loop occurs, mediated by Rad52p^y^ and Rad59p^y^ (RAD52^h^) [26, 74]. Ligation forms a dHJ, which can either be dissolved or resolved by two nucleolytic cuts: symmetrical cuts yield non-crossovers, whereas asymmetric resolution gives crossover products. Repair is thus complete; resulting sequences depend on the HR sub-pathway and whether crossover has occurred, but no indels are created.

### Model for eccDNA formation by HR

Intra-chromatid HR DSBR with crossover presents an intuitive opportunity for eccDNA formation, as the DNA loops in upon itself. Indeed, this model has been proposed in previous studies [2, 8, 75, 76], even before the recognition that an excised region can circularize and be maintained as eccDNA [77–79].

This model only requires a single DSB event, as asymmetric dHJ resolution provides the cuts necessary for circle excision. Single DSBs are more probable than the occurrence of two concurrent DSBs, which we propose are necessary for eccDNA generation through NHEJ and a-EJ.

While SDSA, BIR, and DSBR without crossover could generate eccDNA, this would require two DSBs around extensive homology to create a free fragment for circularization. We regard the probability of both conditions co-occurring as exceedingly rare and the corresponding eccDNA generation as likely to be negligible.

**Figure 3.**
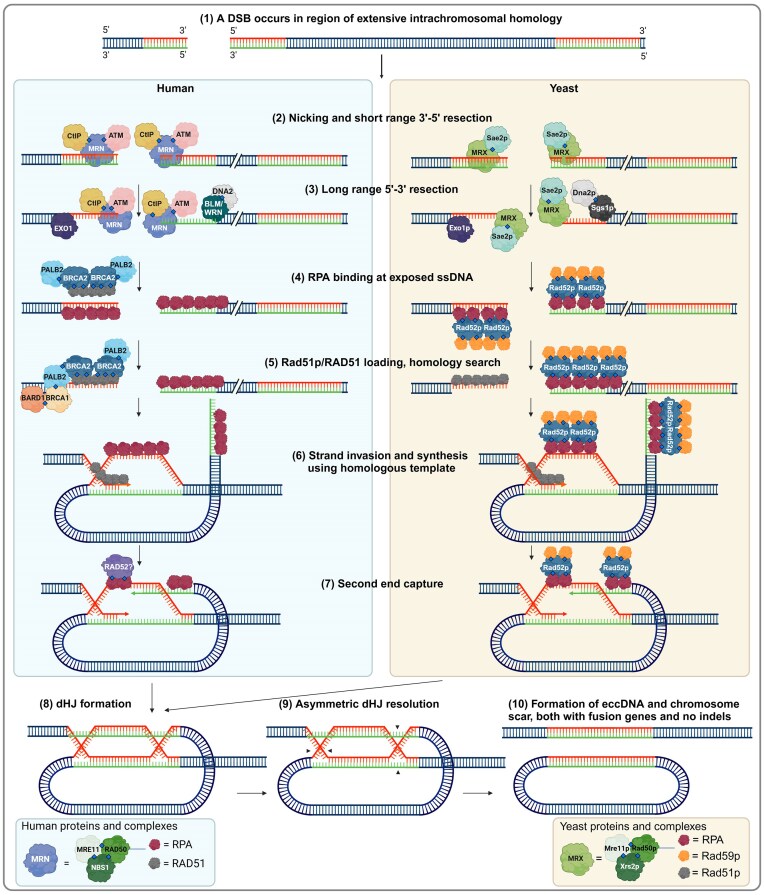
Proposed model for eccDNA generation through HR DSBR, based on models from literature [2, 8, 75, 76] and protein interactions occurring during regular HR DSBR [29, 33]. Diamonds indicate experimentally verified protein interactions. The positioning of the proteins is meant to illustrate where they might be located. The two interacting sequences are homologous genes in direct repeat, e.g. *HXT6* and *HXT7* in *S. cerevisiae*. Arrows indicate steps in the model. Created in BioRender. Eugen-Olsen, R. (2025) https://BioRender.com/x43s014.

Figure 3 demonstrates the proposed mechanism for HR DSBR with crossover and eccDNA generation.

### Evidence for HR in eccDNA generation

Specific paralogous chromosomal sequences are known to circularize with high frequency and are detected as eccDNA in yeast [5], *Drosophila melanogaster* (fruit fly) [76], and *Oryza sativa* (rice) [8]. This suggests that homology between the chromosomal regions plays a crucial role in this eccDNA formation mechanism, as seen in yeast, wherein examples of recurrently circularizing genes encode the hexose transporters (*HXT6* and *HXT7)*, the copper sequestration proteins (*CUP1-1* and *CUP1-2)*, and the sodium transporters (*ENA1*, *ENA2*, and *ENA5)* [5, 18, 75, 80]. All these circles stem from paralogous gene pairs which contain an abundance of homology to act as substrates for HR. Therefore, we propose that such loci may circularize by HR, usually following a single DSB, yielding e.g. an [*HXT6/HXT7^circle^*] and a chromosomal *HXT6/HXT7* fusion gene from the *HXT6 HXT7* locus (Fig. 3).

In addition to paralogues genes, long terminal repeats (LTRs) may form eccDNA, as shown by Gresham *et al.* in yeast [2], wherein the LTRs *YKRCδ11* and *YKRCδ12* can excise a circle carrying the *GAP1* gene, which is correspondingly deleted from the chromosome. Sequencing of the chromosomal deletion and the [*GAP1^circle^*] revealed patterns of resection typical of HR [2]. Additionally, DNA fragments containing tandem repeats have been demonstrated to form eccDNA in pre-blastula embryos of *Xenopus laevis* (frog) [81].

Cohen *et al.* showed that sequences from alpha satellite repeats (structures of ≈170 bp) were overrepresented on eccDNA across multiple human cell lines [82]. Genes in tandem repeat have likewise been found to be overrepresented in a study of fruit fly eccDNA. However, deleting the HR-associated genes *Dmblm* and *okra* did not alter eccDNA levels [76]. Studies have shown a range of repetitive elements, such as satellites and tandem repeats, present in eccDNAs in HeLa cells, human fibroblasts, and other mammals such as mice, rats, hamsters, and monkeys [83].

A study investigating eccDNA in tumors of 80 urothelial bladder carcinoma patients revealed a positive correlation between the expression levels of several DNA repair genes (*LIG3, POLQ, BRCA1*, and *BRCA2)* and the number of eccDNAs per million reads. BRCA1 and BRCA2 are involved in HR, indicating their potential involvement in tumor eccDNA generation [84]. However, concrete evidence for HR-mediated eccDNA formation in humans has not been established. This could also be because in human soma, HR is restricted to actively mitotic cells, rendering it less frequent compared to yeast. Most human cells, including muscles, heart, and neurons, are postmitotic and rely predominantly on NHEJ for DSBR. Consequently, in these cells, HR may not play a significant role in the generation of eccDNA. However, we anticipate that HR mechanisms will contribute to the generation of eccDNA in cancer cells and healthy diving cells, where HR is active. A notable exception is the commonly observed phenomenon of tumors defective in HR [85], in which it remains to be experimentally shown whether eccDNA formation is altered. This represents a key question, because differences in formation rate will affect the genetic variation in cancer cells and thereby the evolution of relapse and chemotherapy resistance.

A recent study in human cells found that microsatellite BIR can generate direct repeats, which can then circularize via intrachromatid HR to generate eccDNA [86]. It was furthermore found that Rad51 depletion and replication stress affected eccDNA mutagenesis.

Besides its role in DNA repair, HR plays a key role in generating genetic diversity during meiosis. eccDNA have recently been found in the pollen of *Amaranthus palmeri* [87] and human sperm cells [13], showing that circles are created and maintained in germline cells. However, no evidence currently suggests that HR forms these circles. Rather, the recombination rate of human chromosomes in meiosis has shown an inverse correlation with eccDNA formation [13], suggesting a different formation mechanism. Despite this current lack of experimental evidence, we propose that the formation of eccDNA through intrachromatid meiotic recombination could play a role in explaining the discrepancies observed between deletions from meiotic intrachromatid recombination and their lack of associated chromosomal amplifications associated with common human congenital disorders [77].

### Alternative end joining

a-EJ encompasses multiple DNA repair mechanisms, many of which rely on microhomology, including MMEJ in yeast and TMEJ in humans [88]. While we have adopted this terminology, there lacks scientific consensus on the nomenclature of a-EJ, partly because TMEJ was coined recently [36, 89]. Some sources make no organismal distinction and refer to both mechanisms as either MMEJ, a-EJ, or alternative NHEJ. However, TMEJ is exclusively used for humans and never for yeast. In this review, the term a-EJ is specifically used to describe MMEJ in yeast and TMEJ in humans.

a-EJ involves the annealing of microhomologous ssDNA. It causes DNA between the microhomologies to protrude as flaps, which are then enzymatically removed (Fig. 1), creating deletions. Because a-EJ requires resection to reveal microhomologies, it shares cell cycle windows with HR, being active in the G2 and S phases where resection is uninhibited [36], but in contrast to HR, human a-EJ remains active in M phase [90]. The essentiality of a-EJ becomes pronounced in HR-deficient cells. However, the extent to which it primarily functions as a backup mechanism to HR and its role in DSBR in cells with intact HR and NHEJ remains unclear. The purpose of human a-EJ might be to repair breaks that contain overhangs or have been resected, rendering them unsuitable for NHEJ, but where an appropriate template for HR is also unavailable [89, 91]. a-EJ always leads to deletions and is thus inherently mutagenic, making it distinct from HR and NHEJ. Furthermore, human a-EJ often introduces insertions at the repair site [89].

a-EJ in yeast was discovered after NHEJ and HR [92] and is less defined [93]. It requires far shorter homology than HR, with estimates around 2–25 bp [30, 35, 94].

Human a-EJ differs from yeast through the essential role of DNA polymerase theta (Polθ), which has no yeast orthologue. Human a-EJ can use shorter microhomologies than yeast a-EJ: while 3 bp of homology in human a-EJ is usually required for annealing, several rounds of synthesis can enable repair using even lower homology [36, 89].

### a-EJ mechanism and proteins involved

Following a DSB, a-EJ starts similar to HR with DNA nicking and limited 3′–5′ resection by Sae2p^y^ and Mre11p^y^ [30] (MRN^h^-CtIP^h^) [36]. If microhomologies are ≤2000 bp apart, this ssDNA exposure is adequate for yeast a-EJ [30]. If further apart, extensive ssDNA may be generated via long-range 5′–3′ resection by Exo1p^y^ (EXO1^h^) and Dna2p-Sgs1p^y^ (DNA2-BLM/WRN^h^) to reveal microhomologies for yeast a-EJ, though it is unclear whether human a-EJ can occur after extended resection [30, 36].

In human a-EJ, PARP1^h^ facilitates Polθ recruitment [95] and aids 5′–3′ nick resection, channelling repair towards a-EJ and possibly HR [96]. Microhomologies between the resected ends are recognized and annealed. This is mediated by Polθ^h^ in human a-EJ [36], while Rad52p^y^ acts as a mediator of ≥15 bp microhomology annealing but an inhibitor of shorter length annealing in yeast a-EJ [97]. Depending on resection length, accordingly sized 3′ DNA flaps protrude from annealed sequences. These flaps are cut off by the Rad10^y^/Rad1^y^ endonuclease (XPF-ERCC1^h^ or Polδ^h^) [36, 98]. After flap removal, gaps are filled by the synthesis of new DNA done by Polδ^y^ in yeast [97], while Polθ^h^ performs initial synthesis in humans, followed by a switch to the more precise Polδ^h^ [99]. After synthesis, DNA is ligated, with the responsible proteins being unclear. Hypothesized mediators are Lig1p^y^ and Dnl4p^y^ in yeast [30, 35, 100, 101], with human candidates being LIG3-XRCC1^h^ and LIG1^h^ [102]. After DNA ligase has sealed the nicks, repair is complete. a-EJ always yields a deletion corresponding to the removed flaps. Human a-EJ furthermore often introduces characteristic insertions [30, 36, 89], which are rarely observed in yeast a-EJ [30].

### Model of eccDNA formation by a-EJ

**Figure 4.**
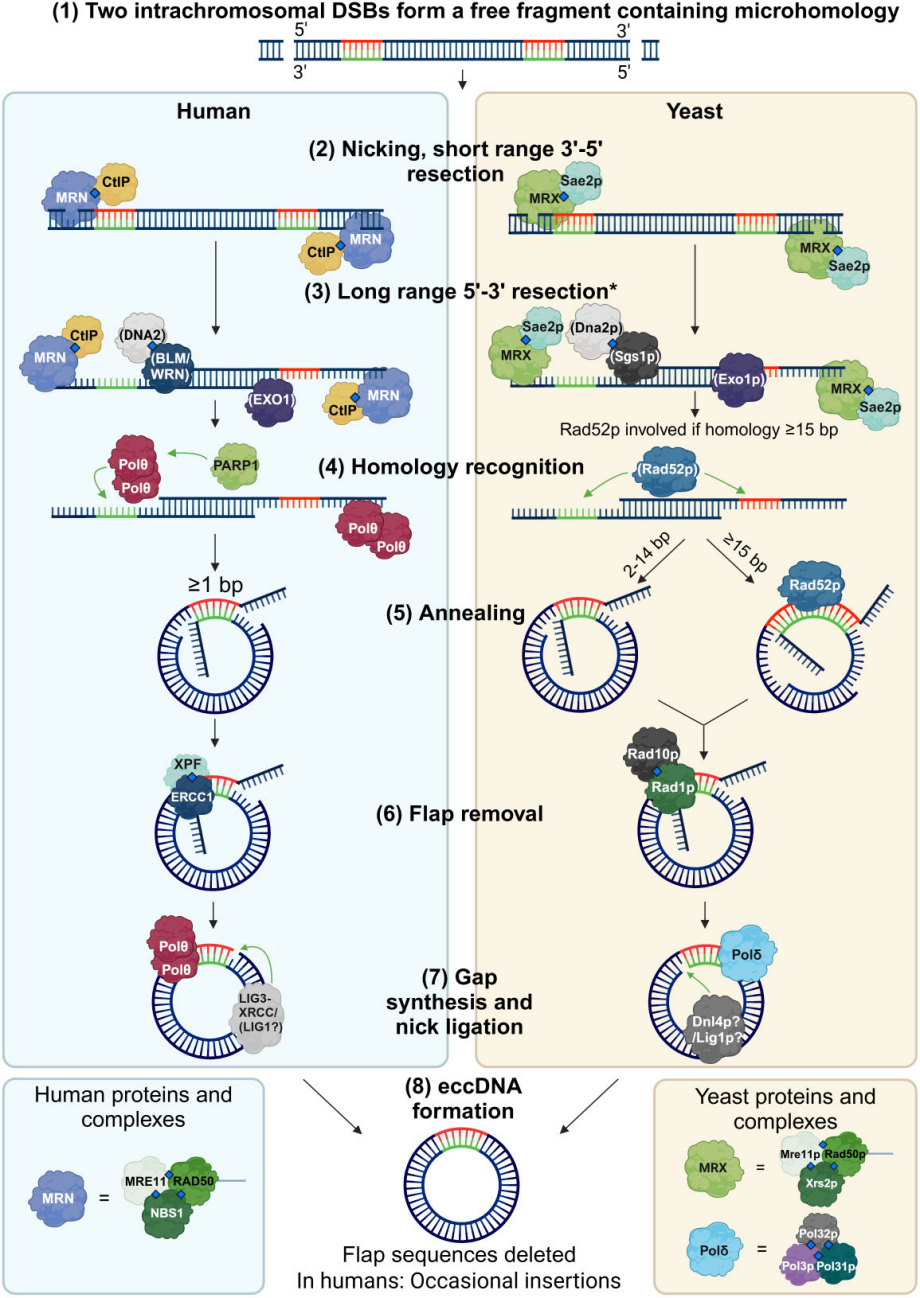
Proposed model for eccDNA generation through a-EJ, based on a model from literature [22] and the protein interactions occurring during regular a-EJ repair of DSBs. Diamonds indicate experimentally verified protein interactions. The positioning of the proteins is meant to illustrate where they might be located. Arrows indicate steps in the model, and protein involvement. In yeast a-EJ, long range resection depicted in step [3] is only required if microhomologies are ≤2000 bp apart. Created in BioRender. Eugen-Olsen, R. (2025) https://BioRender.com/i63q831.

Similar to our NHEJ model, we propose that eccDNA formation through a-EJ requires two concurrent intrachromosomal DSBs, creating a free third fragment (Fig. 4). This can either undergo short or long-range resection to reveal microhomologies, which can ligate to form a circle with protruding flaps. This microhomology-based model has previously been proposed in the context of <400 bp eccDNA formation [22]. Flap removal is followed by gap-filling, DNA ligation, and formation of an eccDNA. The chromosome can be repaired through a DSBR mechanism corresponding to its homology. The flaps cut from the eccDNA will neither be present on the circle nor on the repaired chromosome, and the chromosome will, therefore, contain a deletion corresponding to the eccDNA and the removed flaps.

### Evidence for a-EJ in eccDNA generation

Paulsen *et al.* [24] compared levels of ≤1000 bp eccDNA in human U20S cell lines carrying deletions in DNA repair proteins by quantifying eight known recurring eccDNAs using real-time polymerase chain reaction (qPCR). Cells carrying deletions of NHEJ components had increased eccDNA formation, and deletions of resection-based repair genes involved in a-EJ showed decreased eccDNA loads. Furthermore, eccDNA formation was found to depend on the cell cycle and be heightened during the S, G2, and M phases where a-EJ is active. This could indicate that a-EJ is the main formation mechanism of small human eccDNA, with NHEJ acting as an inhibitory pathway. However, it is unclear whether these results are explained by NHEJ suppressing eccDNA formation in itself or whether NHEJ deficiencies drive repair towards increased a-EJ activity and corresponding a-EJ mediated circle formation, as a-EJ is known to have compensatory activity when NHEJ is compromised [30]. The study furthermore found that a single site-induced DSB was sufficient to elevate microDNA levels, leading the authors to hypothesize an a-EJ-based eccDNA formation method generating single-stranded eccDNA from a single DSB [24]. While single-stranded eccDNA has been observed through electron microscopy [103], models of single-stranded circle formation remain speculative.

The potential ability for human a-EJ to generate eccDNA from only a single DSB suggests that the pathway may be highly efficient at generating small eccDNAs compared to other repair mechanisms, making it an intriguing avenue for further exploration in understanding the dynamics of eccDNA formation. However, investigations are required to identify whether this mechanism could generate large gene-carrying eccDNAs, and whether the observed effect of pathway abrogation on eight specific recurring eccDNAs [24] is generalizable to small eccDNA formation in general.

Links between human a-EJ factors and eccDNA formation have also been observed in patient samples. In a study by Lv *et al.* on eccDNA in urothelial cancer patients, a positive correlation was found between the expression levels of *POLQ* (gene coding for Polθ) and the amount of eccDNA per million reads, revealing a potential link between human a-EJ and eccDNA generation in tumors [84].

a-EJ has been proven important in mobilizing specific retrotransposons that rely on circular intermediates. A recent study found that in fruit fly oocytes, RNA interference (RNAi) silencing of the a-EJ factors Lig3, XRCC1, Fen1, and Polδ all caused decreased eccDNA formation from the *HMS–Beagle* retrotransposon, whereas this was not observed when depleting HR and NHEJ factors. Furthermore, silencing Polδ and XRCC1 caused a more than tenfold reduction in *HMS–Beagle* integration events in the genome. Depleting Lig3, XRCC1, and Fen1 in pupae prevented the *mdg4* retrotransposon from circularizing. These findings were corroborated in mice, where the use of CRISPR to target a-EJ factors Lig3, XRCC1, and Polθ all blocked eccDNA biogenesis from the IAP retrotransposon [104].

There is evidence to suggest that a-EJ could play a role in eccDNA formation in the context of apoptosis. Wang and colleagues [105] demonstrated that during apoptosis in human cell lines, endonucleases degrade DNA, forming DNA fragments that can subsequently ligate to generate eccDNA. They identified the LIG3 protein as a central player in this process of eccDNA formation. The study furthermore demonstrated that eccDNA released to the bloodstream during apoptosis could stimulate the immune system via the STING pathway if circles are transfected to the cytosol. However, it remains unknown whether extracellular eccDNA released during apoptosis is able to enter living cells *in vivo*.

Besides pathway perturbation studies, multiple instances of indirect evidence for a-EJ eccDNA formation exist in microhomology observed at chromosomal sites donating eccDNA flanks. A study of eccDNA in *Bombyx mori* (silkworms) found that short dual direct repeats were commonly found in the chromosomal donor site of eccDNAs and that this pattern was especially prevalent for eccDNAs of below 1000 bp, corroborating the hypothesis that microhomology plays a vital role in the formation of small eccDNAs [106]. Microhomology has likewise been observed at the chromosomal donor sites of small eccDNAs in cancer cells [107]. Furthermore, a study investigating eccDNA content in sperm of humans and mice showed that microhomology was overrepresented and found around most eccDNA breakpoints, indicating that a-EJ could be the main contributor of meiotic eccDNA in higher eukaryotes [108].

### Model limitations and other sources of eccDNA

All canonical DSBR mechanisms for circular DNA formation proposed in Figs 2–4 are predicted to lead to a chromosomal deletion of the sequence forming the eccDNA [10]. eccDNA formation with corresponding chromosomal deletions have been experimentally observed in yeast and human cells [2, 3]. However, eccDNA are not always followed by a detectable deletion [18].

**Figure 5.**
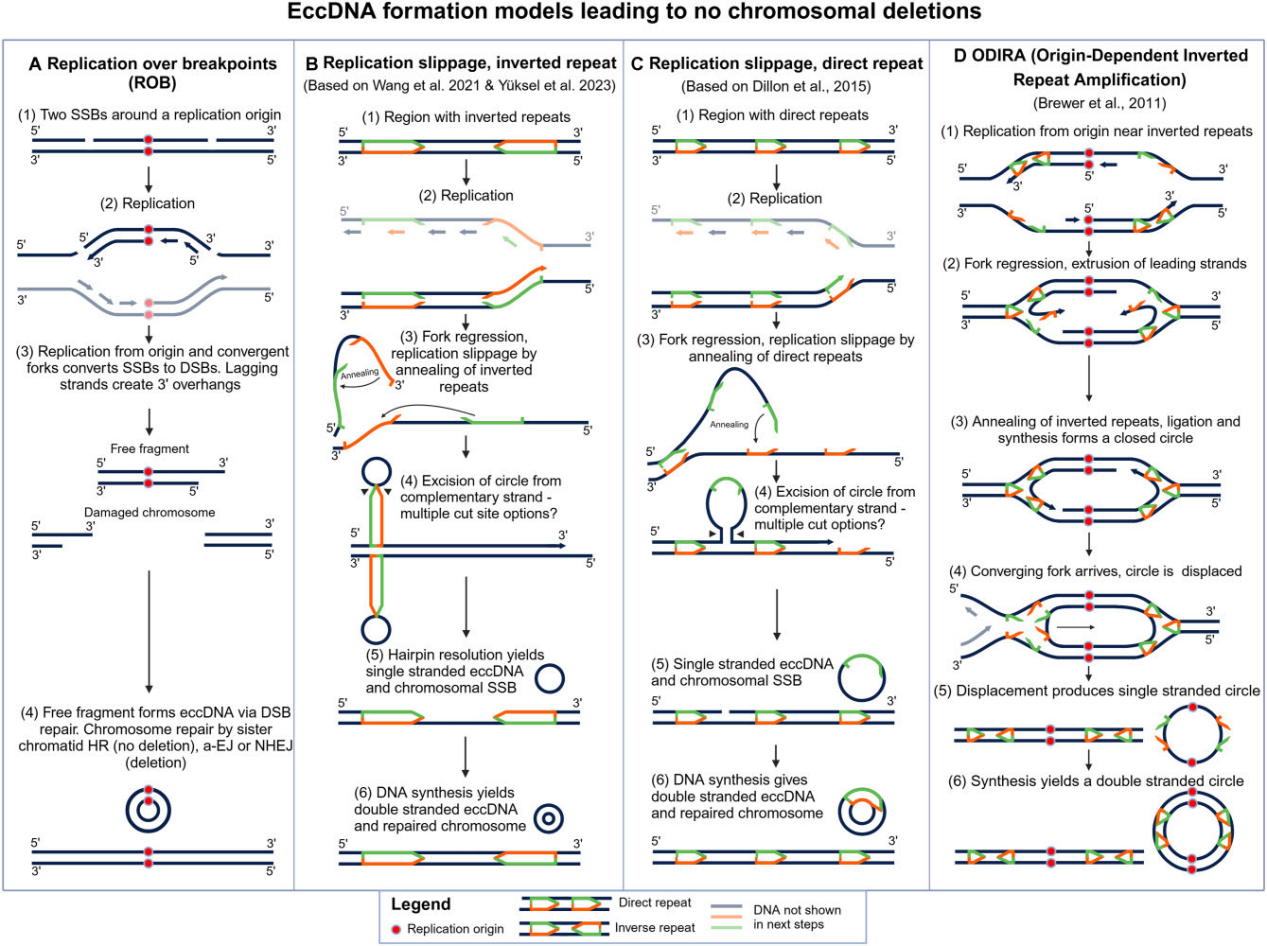
Alternative models of eccDNA formation which do not lead to chromosomal deletions. Dots indicate replication origins. Arrows in the DNA indicate homologous regions and arrowheads indicate their direction. DNA in faded colours in steps (**A**) (2), (**B**) (2), (**C**) (2), and (**D**) (4) are left out of subsequent steps for simplicity. (**A**) Two concurrent SSBs surrounding a replication origin are converted to two DSBs during replication, which can then form an eccDNA through DSBR [Replication Over Breakpoints (ROB)]. (**B**) Replication slippage of inverted repeats (IRs) forms a protrusion of annealed IRs and an unannealed intermittent sequence. If this protrusion is cut from the template strand to form an eccDNA, no chromosomal deletion will occur. However, it is currently not known where the protrusion will be cut. (**C**) Replication slippage of direct repeats forms a protrusion. If this protrusion is cut from the template strand to form an eccDNA [22], no chromosomal deletion will occur. However, it is currently not known where the protrusion will be cut. (**D**) Origin-Dependent Inverted-Repeat Amplification (ODIRA) [25] can form a closed loop of DNA during replication of IRs. When a convergent replication fork approaches, the closed loop can be excised as an eccDNA. Created in BioRender. Eugen-Olsen, R. (2025) https://BioRender.com/g37a424.

**Figure 6.**
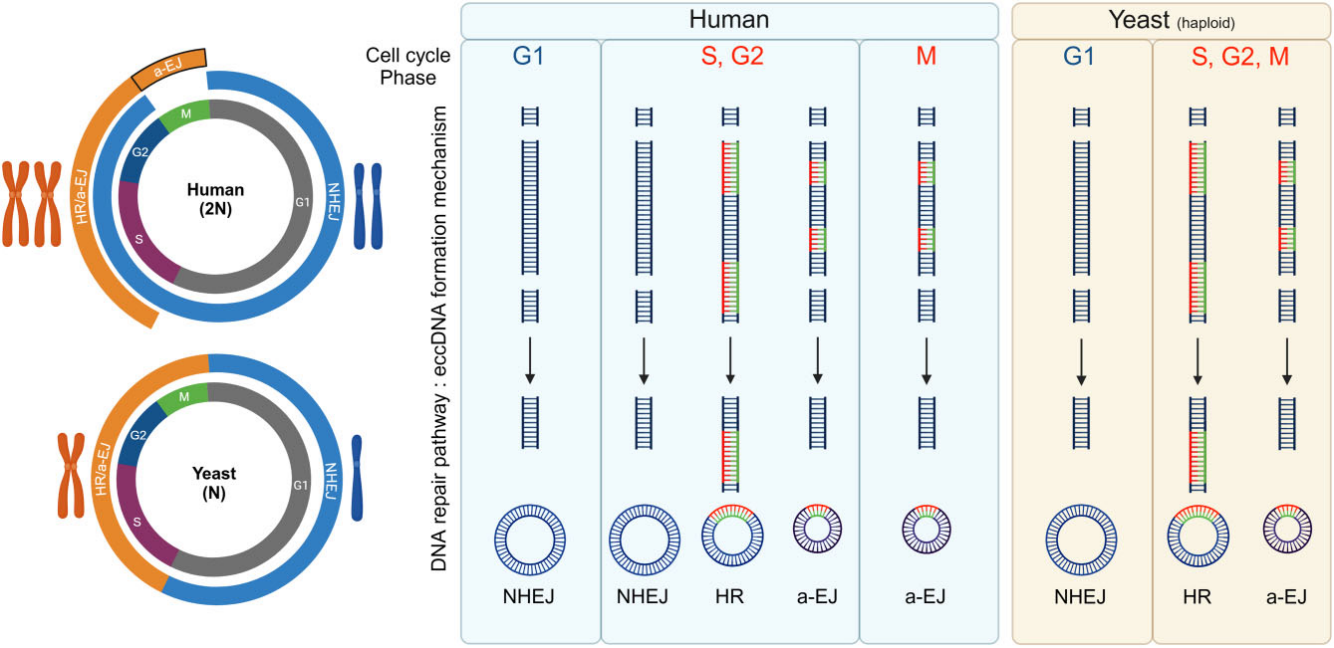
Model of cell cycle stage effect on eccDNA formation. We propose that eccDNA generation by DNA repair depends on the cell cycle stage, as different repair mechanisms dominate during different cell cycle stages. Left: In humans, HR is mainly active during S and G2 phase, while a-EJ is active during S and G2 and remains active during M phase. Human NHEJ is active in all except for M phase. In haploid yeast, NHEJ is only active in G1 phase, while HR and a-EJ are active in S, G2, and M phase. Right: We propose that eccDNA generation will correspond to the DSBR pathways which are active during the given cell cycle phase. Therefore, we expect haploid yeast in G1 phase to form eccDNA through NHEJ, and for haploid yeast in S, G2, and M phase to form eccDNA through a-EJ or HR. In humans, we expect cells in G1 phase to form eccDNA through NHEJ, cells in S and G2 phase to form eccDNA through S and G2 phase, and for cells in M phase to form eccDNA through a-EJ. Created in BioRender. Eugen-Olsen, R. (2025) https://BioRender.com/p97j666.

There could be multiple explanations for the observation of eccDNA in cells that do not have a corresponding chromosomal deletion (Fig. 5). Any of the DSBR based eccDNA formation mechanisms proposed in Figs 2–4 could have the potential to form an eccDNA without a chromosomal deletion. We propose that this situation would require the eccDNA to arise from a chromatid arm on a replicated chromosome in late S, G2 or M phase, after which the eccDNA would have to segregate with the intact sister chromatid during cell division, resulting in a daughter cell with an intact chromosome and an eccDNA, and another daughter cell only carrying the chromosomal deletion. This model would apply to eccDNA formation through DSBR mechanisms active in S, G2, and M phase, which is HR, NHEJ, and TMEJ in humans, and HR and MMEJ in yeast (Fig. 6).

Single-strand breaks (SSBs) are the most common form of DNA damage, occurring at the scale of tens of thousands of breaks in an average cell per day [109]. eccDNA formation is not limited to DSBR but could also result from repair of less severe types of DNA damage, such as SSBs, and repair by the MMR pathway [22, 24, 110], which can generate small eccDNAs. However, it is unclear whether MMR directly generates eccDNAs or whether replication across the SSB leads to a DSB, and the corresponding DSBR leads to eccDNA generation.

Many endogenous DSBs occur during DNA replication, and it is thus relevant to consider eccDNA formation in the context of replication. It has been suggested that a stalled replication fork due to a SSB or other disruption could lead to the nascent DNA strand annealing to itself through microhomology, which when excised could produce a single-stranded circle that could be made double-stranded through synthesis [22]. Replication of a SSB can result in a DSB [111], but to our knowledge, no current eccDNA formation model integrates DSBs arising from replication. Here, we propose that much more common SSBs [109] could lead to concurrent DSBs during replication (Fig. 5A), thus providing substrates for eccDNA formation through DSBR. Leading strand replication across a SSB and the following fork collapse can result in a single ended DSB, whereas lagging strand SSB replication can yield a double ended DSB [111]. If a replication origin is present between the SSBs, subsequent replication can result in two DSB that form an eccDNA, which also carries the capacity to replicate. We call this model ROB (Fig. 6A). Though not confirmed, it seems likely that eccDNAs form frequently from SSBs, because these are much more common than DSBs in genetically normal cells. Besides explaining how eccDNA can form at high rates, the ROB model could also explain the occurrence of eccDNA without reciprocal deletions on the chromosome, as we propose that the deletion caused by the excised circle can be faithfully repaired through sister chromatid HR.

IR sequences have been found near eccDNA donor sites in multiple studies [8, 112, 113], suggesting that they could play a role in eccDNA formation. One potential mechanism could be through the replication slippage of IRs [114] creating protrusions of annealed sequences and an intermittent unannealed loop (Fig. 5B). If the loop is excised from the template strand and then ligated, an eccDNA could form and the chromosome could be repaired to show no corresponding deletion. IR replication slippage models have been proposed in previous reviews [115, 116]. An eccDNA formation model based on replication slippage of direct repeats has also been proposed to explain the formation of eccDNA from DNA repeats without chromosomal deletions [22] (Fig. 5C).

eccDNA can potentially also form without DNA damage and chromosomal deletions through ODIRA [25] (Fig. 5D). In this model, replication fork regression and extrusion may lead to strand reannealing at short IR sequences at the opposite side of the replication bubble. This results in leading strands catching up to and annealing with the lagging strands, yielding a closed circular DNA molecule that exits the chromosome as eccDNA [25]. It can later reintegrate at the homologous chromosomal sequence [117]. In multiple studies, IR has been found at eccDNA chromosomal donor regions, suggesting their role in eccDNA formation [5, 112, 113]. The extent to which ODIRA might contribute to this observation needs further investigation.

The Fanconi anaemia pathway could play an important role in the formation of complex eccDNA from micronuclei, and could therefore be an important source of eccDNA in diseases where micronuclei are observed, such as cancer. In a recent study [118], the FANCI-FANCD2 heterodimer was found to facilitate SLX4-XPF-ERCC1 mediated nucleolytic cleavage of micronuclear chromosomes. After multiple cleavages have shattered the micronuclear chromosome, a mitotic DNA synthesis process known as MiDAS converts ssDNA ends into DSB ends through POLD3, thus rendering the fragments suitable for assembly into complex circles through NHEJ [118].

SSA [27] is the only DSBR mechanism not included in our models. Briefly, it relies on direct annealing of extensive homologies to produce deletions between the homologous areas, similar to the deletions created in a-EJ. SSA could create eccDNA following two intrachromosomal DSBs when extensive homology is present near both DSB sites, but similar to eccDNA formation through SDSA and BIR, we regard the co-occurrence of both prerequisites to be exceedingly rare, and for corresponding eccDNA generation to likely be negligible. Studies of ≤1000 bp eccDNA have failed to find any connection between SSA proteins and eccDNA load [22, 24], though the same studies also found no link between HR and eccDNA load, indicating that such small eccDNAs are perhaps not formed from extensive homology in general.

## Concluding remarks and future perspectives

In this review, we discuss the eccDNA formation potential of three DNA repair pathways, NHEJ, HR, and a-EJ, and present corresponding models (Fig. 6). Studies indicate that NHEJ can generate eccDNA from across the genome whenever a DNA fragment has been excised by two intrachromosomal DSBs, which can either occur stochastically or accumulate in the context of more significant catastrophic events such as chromothripsis or breakage-fusion bridges. a-EJ may generate eccDNA from the same substrates when they carry microhomologies, and furthermore seems essential in generating eccDNA intermediates from retrotransposons and small eccDNAs (≤1000 bp), though we propose that a-EJ can generate eccDNA of any size. HR is the likely driver of eccDNA generation from paralogous genes in direct repeat in yeast, where it may only require one DSB to induce circularization. Some of these paralogous genes are recurrent in yeast eccDNA screenings, and their ability to reliably mediate copy number changes through intra-chromatid HR plays an essential role in rapid evolutionary adaptation.

Knowledge about the role of DNA repair in eccDNA formation could hold clinical significance. eccDNA can accelerate tumor evolution in cancer [14], and limiting its formation could, therefore, be desirable. Drugs interfering with DNA repair, e.g. PARP inhibitors [119], are already used to treat certain cancers which are deficient in HR and rely on a-EJ for DSBR, and elucidating how such drugs affect eccDNA formation could therefore be relevant to incorporate into treatment strategies. PARP likewise plays a key role in the repair of human SSBs [119]. If the ROB model of eccDNA formation (Fig. 5) is accurate, it could imply further clinical relevance of PARP inhibitors in eccDNA-associated cancer treatment.

The extent to which these pathways contribute to the overall cellular eccDNA pool will necessarily be influenced by the corresponding active repair pathways in the cell, which is determined by the cell type and cell cycle stage of the organism studied (Fig. 6). While many facets of the involvement of each DSBR pathway in forming eccDNA have recently been uncovered, robust study designs are still required to accurately determine the proportional importance of each pathway in different organisms. Due to the inherent randomness in eccDNA formation and the ability of DSBR mechanisms to compensate for one another’s lowered activity, innovative study designs are required to provide conclusive evidence of the involvement of specific pathways.

To investigate the impact of DNA repair pathways on eccDNA formation, one can utilize various perturbation methods to manipulate these pathways and then assess eccDNA levels. Perturbation strategies may include generating single or double mutants, employing RNAi or CRISPR interference to knock down specific DNA repair proteins, and using pathway inhibitors to modulate gene expression. For a valid experimental setup, achieving accurate eccDNA formation rates is critical. An experimental approach following the principle outlined in the Luria–Delbrück fluctuation assay [120] of starting with single cells and growing cell populations to the desired size is advisable, thus minimizing the risk of progenitor cells already harboring eccDNA. This approach ensures that any eccDNA identified in the final cell population formed during cell division, enabling unbiased genotype comparisons.

In conclusion, understanding the intricate mechanisms governing eccDNA formation provides invaluable insights into cellular biology, with significant implications for disease pathology and therapeutic development. Moving forward, continued investigation into eccDNA biology promises to uncover novel therapeutic targets and advance our understanding of disease etiology, ultimately paving the way for improved diagnostic and treatment strategies in cancer.

## Glossary

**Table:** 

Glossary		
Term	Abbreviation	Meaning
**Alternative end joining**	a-EJ	Umbrella term for a variety of less commonly used DSB repair pathways, including MMEJ in yeast and TMEJ in humans.
**Circle-Seq**	n/a	A method for investigating the total eccDNA content of a cell population through DNA column purification, linear DNA removal by exonuclease, and rolling circle amplification of the resulting circular DNA.
**CRISPR-C**	n/a	Technique in which one or more chromosomal DSBs are induced by CRISPR-Cas9 to investigate eccDNA formation from the specific region.
**Direct repeat**	n/a	Sequences present in multiple copies in the genome in the same orientation. Direct repeats on the same chromosome can recombine through intrachromosomal recombination, also known as non-allelic homologous recombination.
**Double-strand break**	DSB	Serious type of DNA damage in which both DNA strands break, resulting in two separate DNA fragments.
**Double-strand break repair**	DSBR	A specific subset of homologous recombination in which double Holliday junctions are formed between the invading strand and its homologous template. Repair products depend on how the junctions are resolved.
**Double-stranded DNA**	dsDNA	n/a
**Extrachromosomal circular DNA**	eccDNA	Umbrella term for eukaryotic circular DNA molecules which are derived from chromosomal sequences.
**Homologous recombination**	HR	Precise DSB repair mechanism which requires lengthy homology near the break. It relies on invasion of a homologous sequence and synthesis of new DNA using the invaded area as a template.
**Inverted repeat**	IR	Sequences present in multiple copies in the genome in opposing orientations.
**Microhomology-mediated end joining**	MMEJ	Type of a-EJ repair found in yeast. Mutagenic DSB repair mechanism which ligates two DNA strands through the annealing of two areas of short homology.
**Nonhomologous end joining**	NHEJ	DSB repair pathway which indiscriminately ligates two DNA molecules together with varying accuracy.
**Origin-Dependent Inverted-Repeat Amplification**	ODIRA	A model for the generation of circular DNA through the annealing of leading and lagging strands during DNA replication.
**Polymerase theta-mediated end joining/Polθ-mediated end joining**	TMEJ	Type of a-EJ repair found in humans. Mutagenic DSB repair mechanism which ligates two DNA strands through the annealing of two areas of short homology.
**Replication Over Breakpoints model**	ROB	Model proposed in this review, in which two SSBs surrounding a replication origin are converted into two DSBs during replication, after which DSB repair forms an eccDNA of the fragment between the DSBs.
**Single-strand annealing**	SSA	Mutagenic DNA repair mechanism which ligates two DNA strands through the annealing of two areas of extensive homology.
**Single-stranded DNA**	ssDNA	n/a
**Single-strand break**	SSB	Common and less serious form of DNA damage in which one stand is broken, but the complementary strand remains intact to serve as a template for repair. Multiple unrepaired single-strand breaks can result in more serious double-strand breaks.

## Data Availability

No new data were generated or analysed in support of this research.
