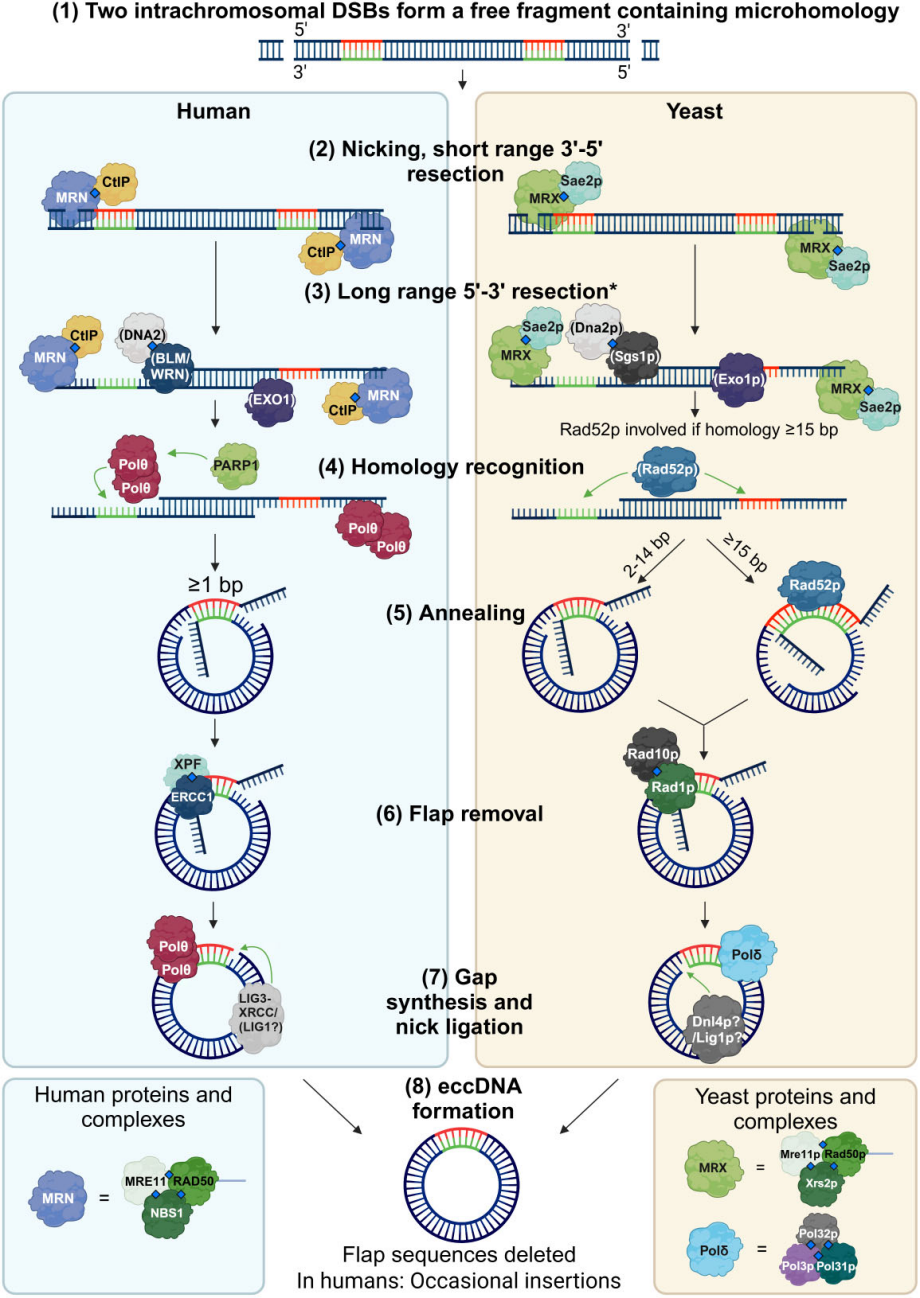# Correction to ‘Molecular mechanisms of extrachromosomal circular DNA formation’

**DOI:** 10.1093/nar/gkaf491

**Published:** 2025-06-04

This is a correction to: Rasmus A B Eugen-Olsen, Judith M Hariprakash, Vibe H Oestergaard, Birgitte Regenberg, Molecular mechanisms of extrachromosomal circular DNA formation, Nucleic Acids Research, Volume 53, Issue 5, 24 March 2025, gkaf122, https://doi.org/10.1093/nar/gkaf122

Errors were inadvertently introduced in Figure 4 during the revision process and after acceptance of the manuscript. Specifically:

- Portions of DNA that were intended to appear in dark blue were incorrectly rendered in red or teal. This issue arose after acceptance, when the authors provided their BioRender license.
- An extraneous “5′” label was mistakenly added above step (3) during revision.

As a result, the BioRender URL for Figure 4 has been updated to: https://BioRender.com/i63q831.

Figure 4 has now been corrected. These changes do not affect the results, discussion, or conclusions of the article. The published version has been updated accordingly.